# Quantified growth of the human embryonic heart

**DOI:** 10.1242/bio.057059

**Published:** 2021-02-10

**Authors:** Jaeike W. Faber, Jaco Hagoort, Antoon F. M. Moorman, Vincent M. Christoffels, Bjarke Jensen

**Affiliations:** Department of Medical Biology, Amsterdam Cardiovascular Sciences, Amsterdam UMC, University of Amsterdam, Meibergdreef 15, 1105AZ, Amsterdam, The Netherlands

**Keywords:** Human heart development, Embryology, Growth, Morphogenesis

## Abstract

The size and growth patterns of the components of the human embryonic heart have remained largely undefined. To provide these data, three-dimensional heart models were generated from immunohistochemically stained sections of ten human embryonic hearts ranging from Carnegie stage 10 to 23. Fifty-eight key structures were annotated and volumetrically assessed. Sizes of the septal foramina and atrioventricular canal opening were also measured. The heart grows exponentially throughout embryonic development. There was consistently less left than right atrial myocardium, and less right than left ventricular myocardium. We observed a later onset of trabeculation in the left atrium compared to the right. Morphometry showed that the rightward expansion of the atrioventricular canal starts in week 5. The septal foramina are less than 0.1 mm^2^ and are, therefore, much smaller than postnatal septal defects. This chronological, graphical atlas of the growth patterns of cardiac components in the human embryo provides quantified references for normal heart development. Thereby, this atlas may support early detection of cardiac malformations in the foetus.

This article has an associated First Person interview with the first author of the paper.

## Abstract (precis)

**Summary:** This paper provides quantitative data on the growth of 58 structures of the human embryonic heart between weeks 4 and 8 of development.

## INTRODUCTION

The study of normal and abnormal growth of individual organs such as the brain, kidney, liver and spleen in the developing human can inform us on disease processes that might become of importance later in life (Latini et al., 2004; Wahab Abdel Latif et al., 2017). For the heart, early diagnoses of malformations allow for better adjusted postpartum care (Thakur et al., 2016) and lead to better survival (Holland et al., 2015). Heart development is a highly complex multistep process. Most comprehensive descriptions of human heart development are qualitative in nature and are summarised in textbooks (Filipoiu, 2014; Oostra et al., 2007; Sadler, 2006; Schoenwolf et al., 2009). These textbooks make use of the many detailed qualitative studies on the emergence and appearance of structures (e.g. Lockhart et al., 2011 and Sylva et al., 2013) or processes like septation (e.g. Anderson et al., 2002; Patten, 1938 and Van Mierop et al., 1963). However, what constitutes normal and abnormal growth of the structures of the human embryonic heart is poorly understood. Nevertheless, in recent years, immunohistochemical studies, regarding for example the development of the cardiac conduction system, have clarified some of the controversies emerging from purely anatomical descriptions (Sizarov et al., 2011a). But, for the single cell transcriptomic data of the developing heart, which is becoming available (e.g. Cui et al., 2019), more detailed knowledge regarding cardiac structures and their sizes may help to validate the assignment of cell identities and their anatomical positions.

The few publications that do address quantitative descriptions of embryonic cardiac growth mostly focus on a detailed point such as ventricular wall thickness (e.g. Goor et al., 1970) or ventricular myocardial volume (e.g. Wenink, 1992). But, most of what is known about quantitative morphology stems from non-human heart development, such as mouse (de Boer et al., 2012; Ishiwata et al., 2003) and chicken (Rana et al., 2007; Rychterová, 1971). No quantitative reference work exists on the growth of key structures of the human heart that can indicate whether cardiac development might be deviating from normal.

Cardiac malformations are usually diagnosed after a (first trimester) ultrasound and/or with an obduction after (spontaneous) abortion (Huggon et al., 2002; Rasiah et al., 2006). With the advancement of visualisation technology, the threshold for detecting abnormalities is shifting to younger stages of development. Since many cardiac malformations are defined by a disproportion and, therefore, by a change in differential growth, a reference work on cardiac growth could be useful. For example, hypoplastic left heart and obstructed foramen ovale as well as septal defects and absence of valvular structures like in tricuspid atresia, result from one structure growing faster or slower than others (Maeno et al., 1999). Additionally, more subtle phenotypes are defined by changes in relative size of myocardial structures, like, for example, left ventricular non-compaction (Menon et al., 2007) in which the proportion of trabecular to compact ventricular myocardium is greater than normal.

Here we present an atlas of normal human embryonic heart development. Given the number of structures investigated, numerous analyses can be made. For the sake of brevity, we have focused on a few processes to exemplify the utility of such quantifications.

## RESULTS

**Fig. 1.**
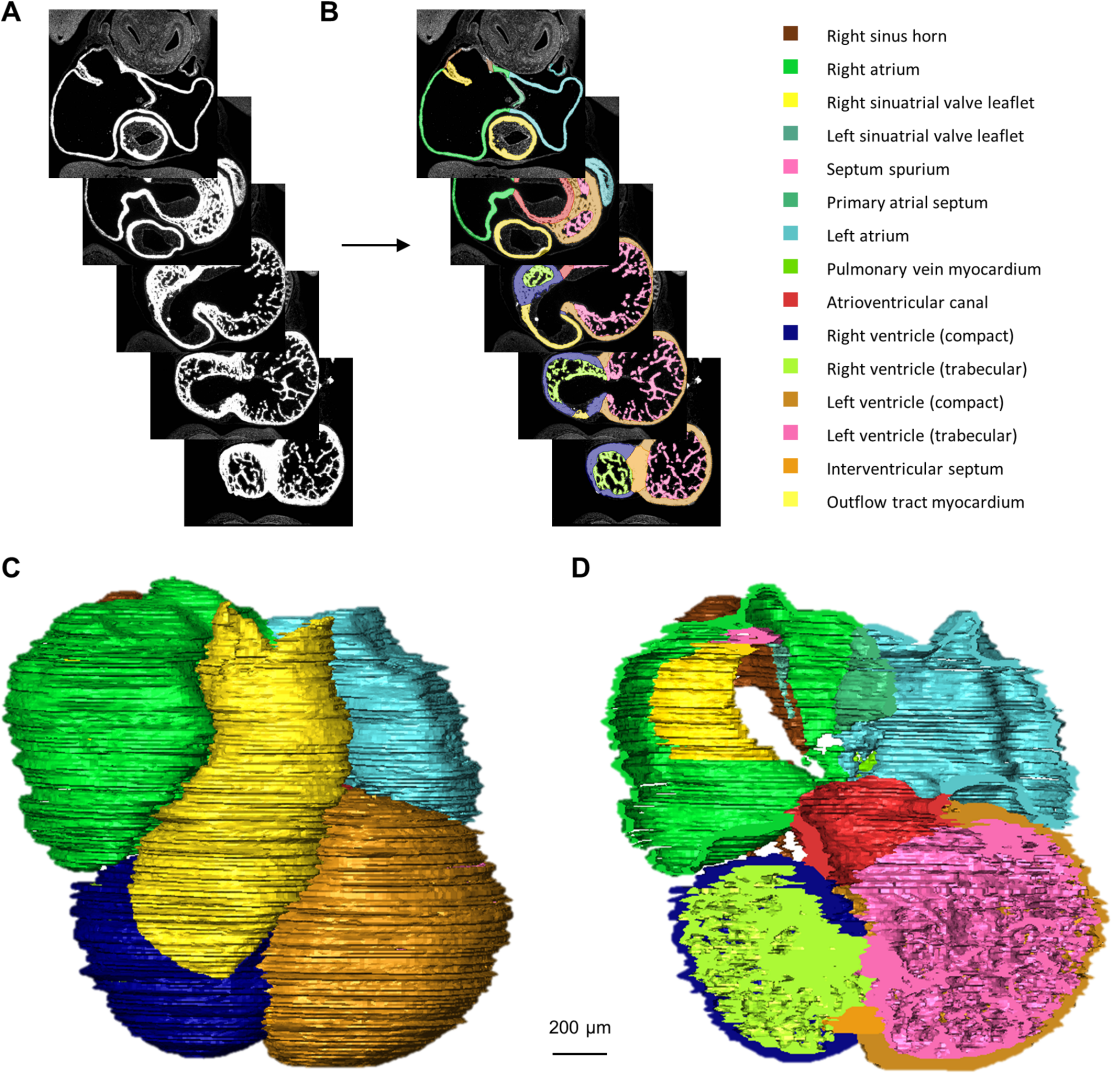
**Methodology.** (A) Sections of the heart were immunofluorescently stained for myocardium and aligned in Amira. (B) Examples of labelling of cardiac structures (cushions and lumina are not shown in this view). (C,D) In Amira, 3D reconstructions can be made of any of the segmented structures, which can be sectioned in any wanted direction (D). Here, the heart of CS14 (*N*=1) was segmented for all listed myocardial structures. No statistical tests were performed.

**Fig. 2.**
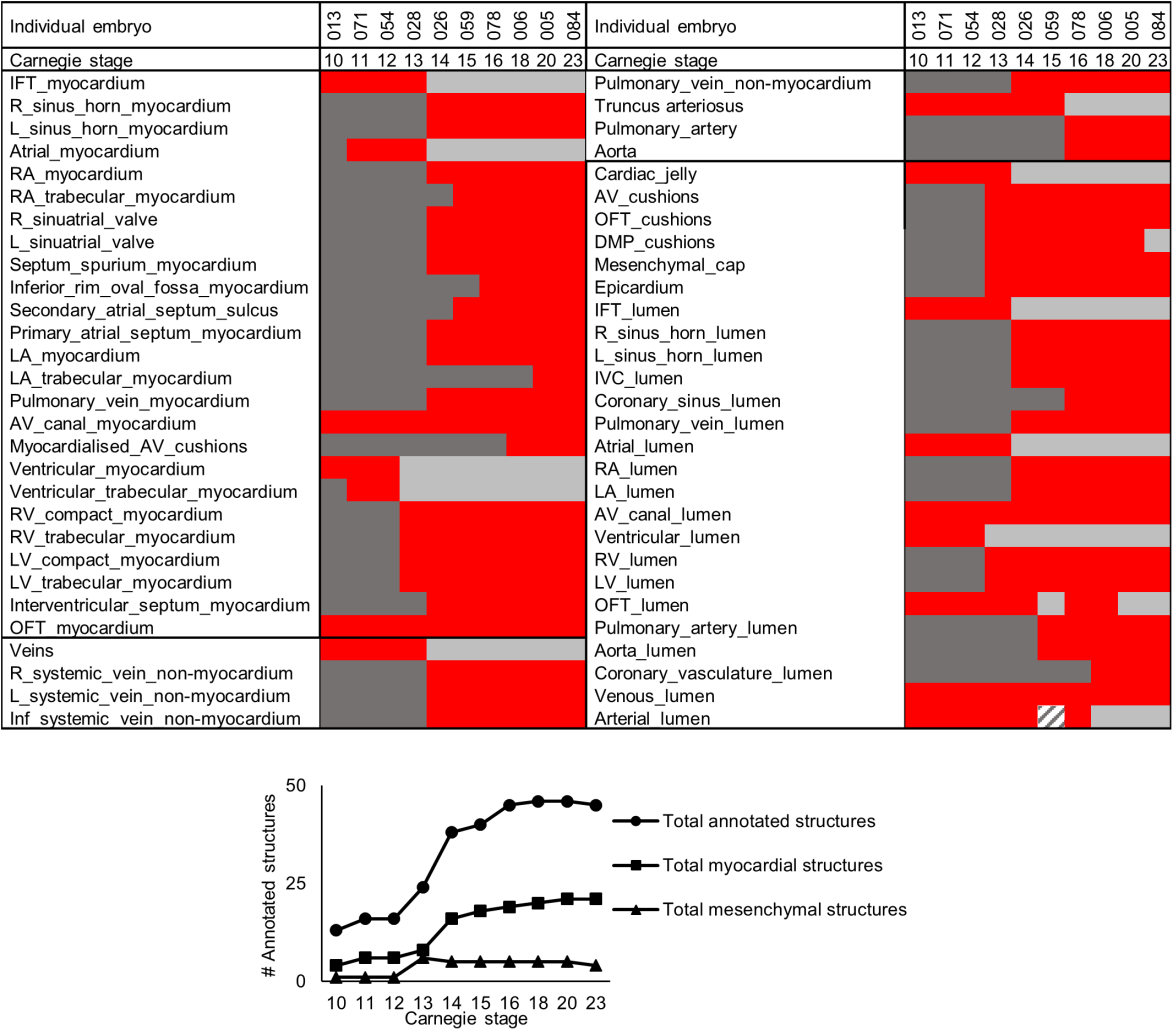
**All structures labelled in each heart.** In red, structures identified on the sections; in dark grey, structures that are yet to emerge and in light grey, structures that have become obsolete due to advanced development, such as the common atrium (*Atrial_myocardium*). The striped box represents a structure that was not on the sections that were included for annotation. *N*=1 for each time point, no statistical tests were performed.

We segmented all major components of ten embryonic hearts of subsequent stages of development (Fig. 1) resulting in three-dimensional (3D) models in which all individual structures can be rotated and inspected (Figshare Supplement). The chronological appearance of the morphologically distinct structures of the embryonic heart have been represented in a graph (Fig. 2). This indicates that most myocardial structures become recognisable during the period of CS12-15, in the fifth week of gestation, which suggests this is a highly critical period for heart development.

### Volumetry

**Fig. 3.**
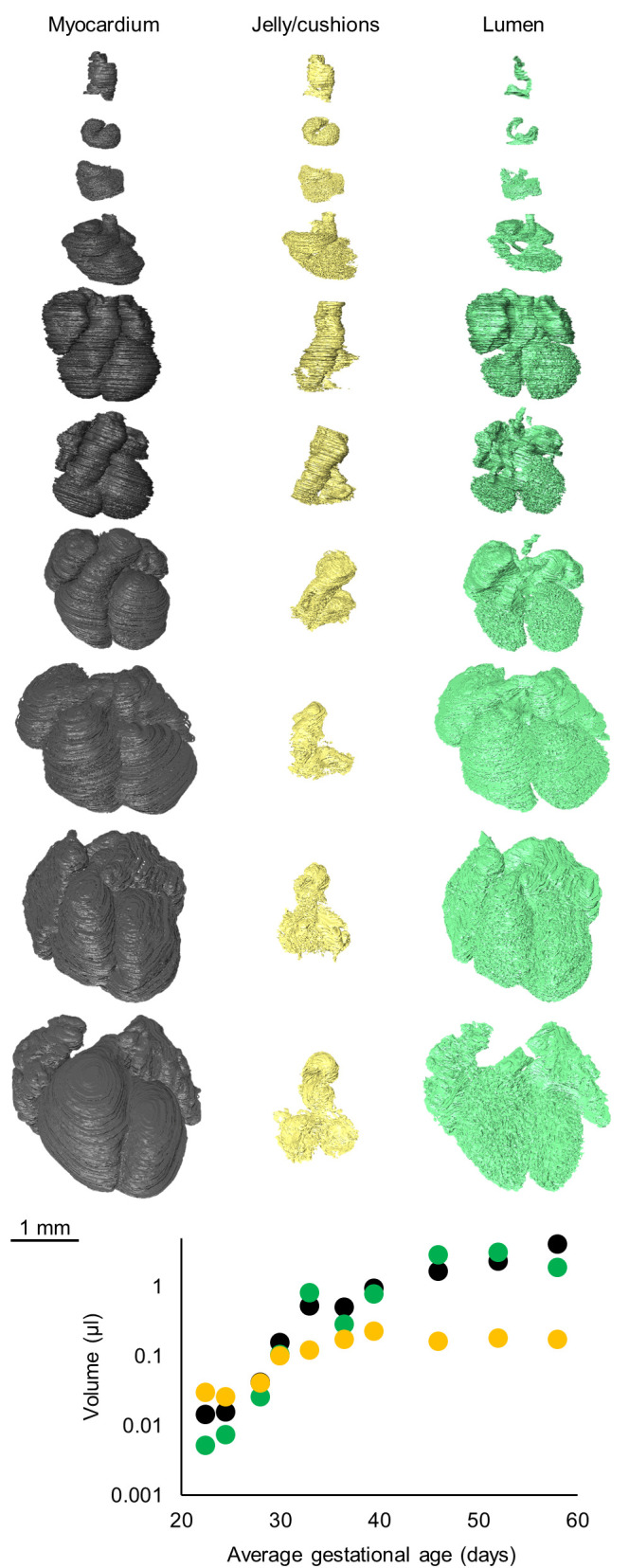
**Growth curves of the myocardium, cardiac jelly or cushions, and lumen of the embryonic hearts.** All myocardial labels (grey), all cardiac jelly or cushion labels (yellow), and the labels for lumen (green) only within myocardial structures, as listed in Fig. 2, were pooled to generate total volumes. There is no indication that the total amount of cushion mesenchyme decreases (logistic regression curve fit R^2^=0.91, *N*=1 for each time point).

Reconstruction of the myocardium, cardiac jelly/cushion tissue, and lumen of the embryonic hearts revealed differences in their relative growth patterns (Fig. 3). The myocardium grows exponentially while the cardiac jelly/cushions slow their growth after CS16. There is no indication that the total amount of cushion mesenchyme decreases in the embryonic period, instead it plateaus after initial growth (logistic regression curve fit R^2^=0.91), which fits with previous reports (Wenink, 1992). The luminal volume increased exponentially over time, although the variance there is larger than for the myocardium, likely because luminal volume is dependent on the state of contraction, which varies among the specimens, whereas the myocardial volume is not.

#### Chambers

**Fig. 4.**
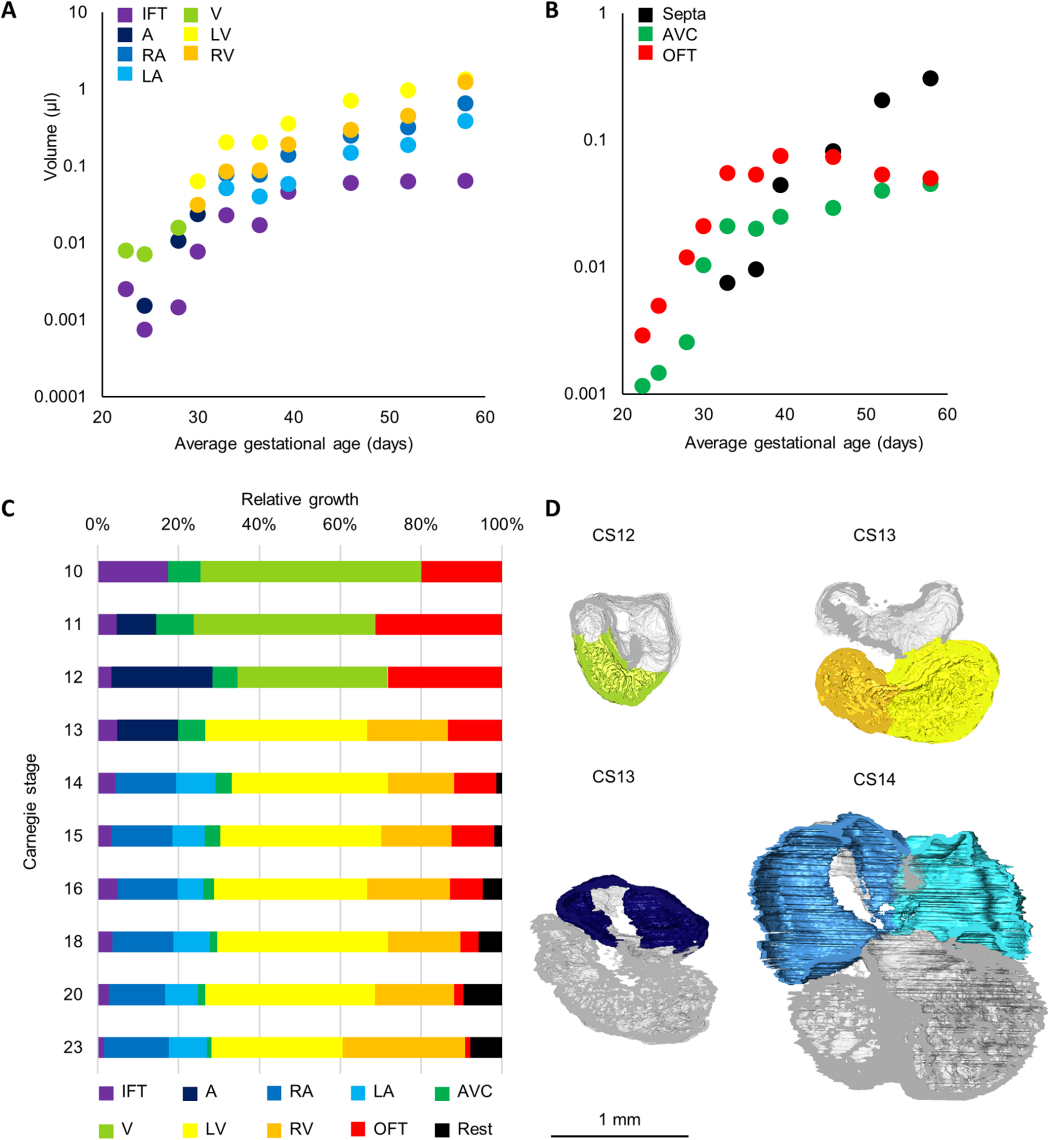
**Growth of the cardiac chambers and myocardial structures.** (A) Absolute growth of the inflow tract (IFT; including *IFT_myocardium*, *R_sinus_horn_myocardium* and *L_sinus_horn_myocardium*), common atrium (A; *Atrial_myocardium*), right atrium (RA; including *RA_myocardium*, *RA_trabecular_myocardium*, *R_sinuatrial_valve*, *L_sinuatrial_valve*, *Septum_spurium_myocardium*, *Inferior_rim_oval_fossa_myocardium* and *Secondary_atrial_septum_sulcus*), left atrium (LA; including *LA_myocardium*, *LA_trabecular_myocardium* and *Pulmonary_vein_myocardium*), common ventricle (V; including *Ventricular_myocardium* and *Ventricular_trabecular_myocardium*), left ventricle (LV; including *LV_compact_myocardium* and *LV_trabecular_myocardium*), and right ventricle (RV; including *RV_compact_myocardium* and *RV_trabecular_myocardium*). There is always significantly less left atrial than right atrial myocardium (*P*<0.0001) and less right ventricular than left ventricular myocardium (*P*=0.002). (B) Absolute growth of the septa (S; including *Primary_atrial_septum_myocardium* and *Interventricular_septum_myocardium*), atrioventricular canal (AVC; *AV_canal_myocardium*), and the outflow tract (OFT; *OFT_myocardium*). (C) Relative growth of chamber myocardium. Rest includes *Primary_atrial_septum_myocardium*, *Interventricular_septum_myocardium* and *Myocardialised_AV_cushion*. (D) Four chamber view cross-sections of hearts of CS12, 13 and 14 illustrating the transition from common atrium (dark blue) and common ventricle (light green) to recognisable right and left atria (intermediate and light blue) and ventricles (orange and yellow). *N*=1 for each time point.

The myocardial growth of the cardiac chambers is outlined in Fig. 4. The inflow tract myocardium, which surrounds the two superior caval veins and may act as a chamber in early human development (Faber et al., 2019), initially grows exponentially, but its growth levels off halfway through embryonic development. It remains to be shown whether this change in growth rate coincides with the so-called atrialisation of this myocardium, whereby the sinus venosus loses its identity as a separate chamber. It can be seen that the amount of right atrial myocardium is always larger than that of the left atrium [non-linear regression, exponential curve fit (R^2^ of 0.98 for both curves), *P*<0.0001]. Conversely, the left ventricular myocardial volume is greater than the right in the entire embryonic period [non-linear regression, exponential curve fit (R^2^ of 0.98 for right and 0.96 for left ventricular myocardial volume), *P*=0.002]. The right ventricular myocardial volume only approaches that of the left ventricle towards the end of the embryonic period. During the first stages of development, the right ventricular growth is driven by the addition of outflow tract myocardium (Kelly et al., 2001; Rana et al., 2007), which is reflected in the drop of outflow tract myocardial volume (Fig. 4B). The atrioventricular canal myocardium initially consists of primary myocardium. This is known to grow very little (Hoogaars et al., 2004; Sizarov et al., 2011b). Therefore, the amount of atrioventricular canal myocardium decreases relatively to the rest of the heart (Fig. 4C).

#### Trabeculae

**Fig. 5.**
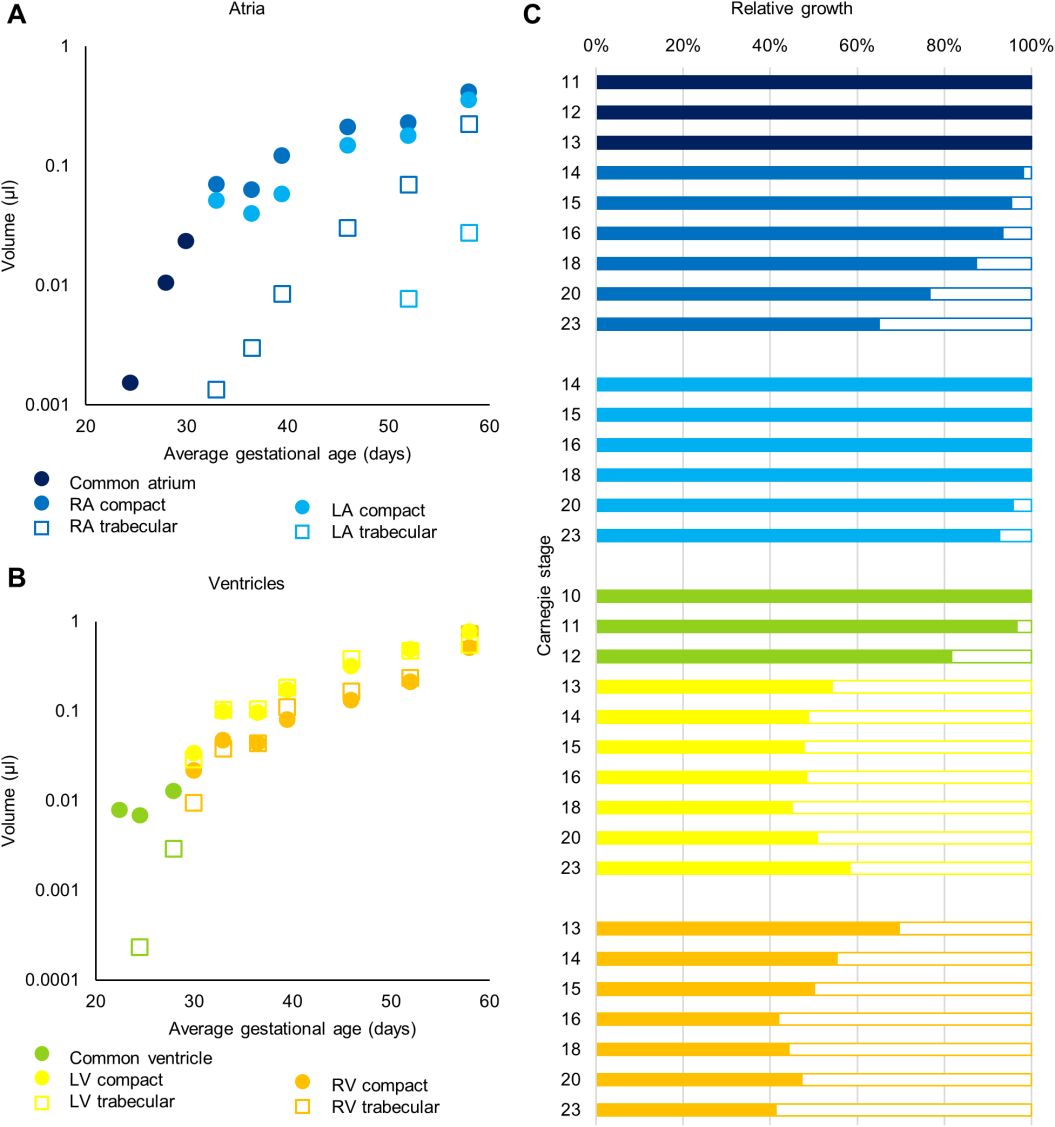
**Atrial and ventricular compact and trabecular myocardial growth.** For the atria, distinction between outer myocardium and the trabecular pectinate muscles is made. For the ventricles the trabecular myocardium, including the papillary muscles, is segmented separated from the compact myocardium. Common atrium (includes *Atrial_myocardium*), right atrium (RA; compact includes *RA_myocardium*, *Inferior_rim_oval_fossa_myocardium* and *Secondary_atrial_septum_sulcus;* trabecular includes *RA_trabecular_myocardium* and *Septum_spurium_myocardium*), left atrium (LA; compact includes *LA_myocardium* and *Pulmonary_vein_myocardium*; trabecular includes *LA_trabecular_myocardium*), common ventricle (includes *Ventricular_myocardium* and *Ventricular_trabecular_myocardium*), left ventricle (LV; compact includes *LV_compact_myocardium*; trabecular includes *LV_trabecular_myocardium*), right ventricle (RV; compact includes *RV_compact_myocardium*; trabecular includes *RV_trabecular_myocardium*). *N*=1 for each time point, no statistical tests were performed.

Both atria and ventricles show development of trabecular structures (see Fig. 1D for examples of the ventricular trabecular labels). In the atria they give rise to the pectinate muscles and in the ventricles they become the trabeculae carneae, papillary muscles and Purkinje network. The distinction between the different trabecular muscle types is more pronounced in adult hearts, where the pectinate muscles also show a lot of individual variation in morphology (Loukas et al., 2008). It can be seen that in the right atrium the trabeculae develop earlier than in the left atrium (Fig. 5A), this is corroborated by data found in several publications (Anderson et al., 2003; Kim et al., 2001; Mandarim-de-Lacerda and Sampaio, 1987; Picazo-Angelin et al., 2018; Wessels et al., 1992, 2000). The left atrium in the embryo is, therefore, smoother than the right atrium, a characteristic that will persist into adulthood (Ho et al., 2002). In contrast to the right ventricle, the left ventricle contains more compact than trabecular myocardium (Fig. 5B,C). The volume of ventricular trabecular myocardium increased for every stage analysed. This contrasts the expectation that a substantial amount of trabecular muscle is added to the compact wall in the process of compaction (Oechslin and Jenni, 2011).

### Morphometry

**Fig. 6.**
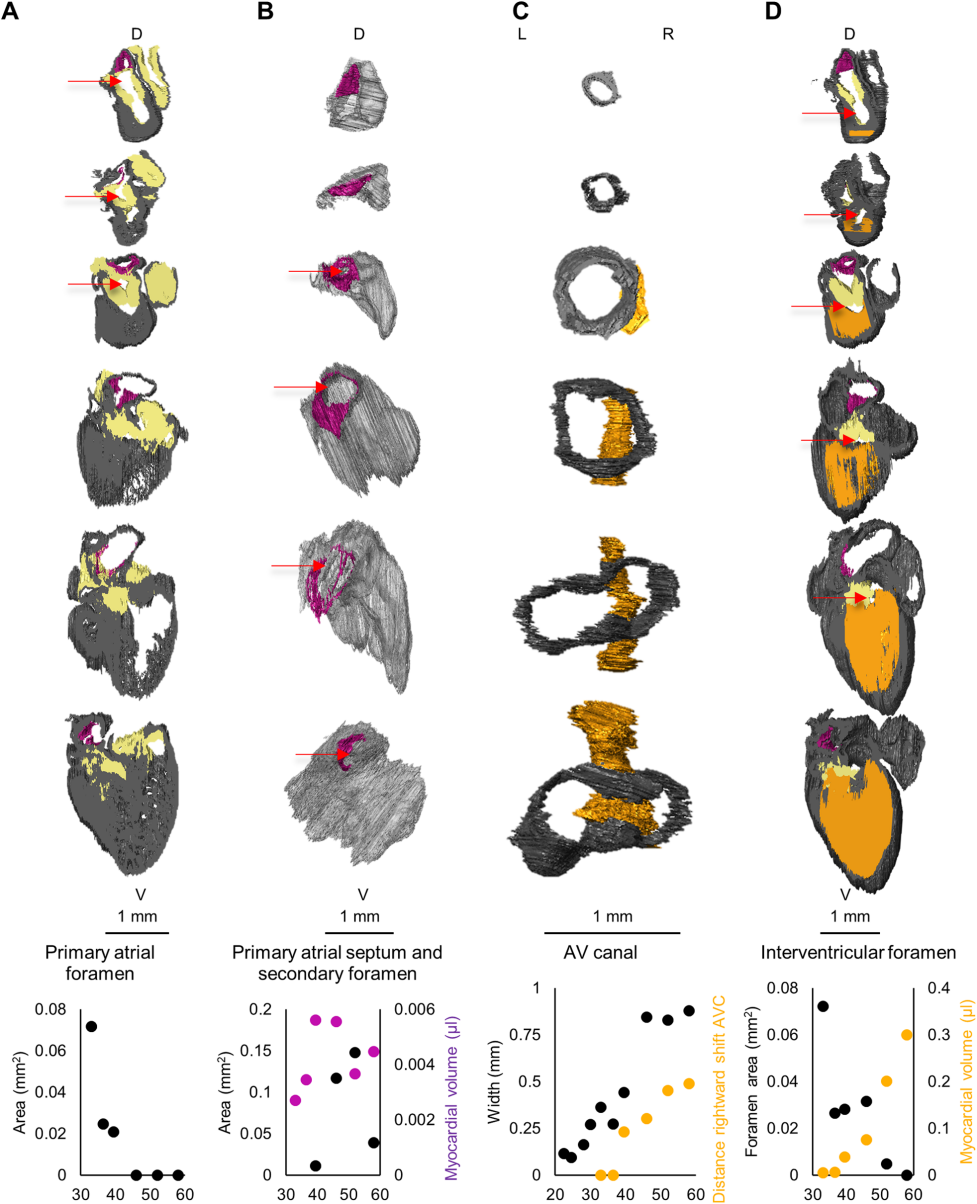
**Morphometric quantifications. (**A) Closure of the primary atrial foramen. In purple, Primary_atrial_septum_myocardium; in grey, all other myocardium; in yellow, cardiac cushions. The angle of cross-section was determined by the primary atrial septum. Slices represent 5% of the total model thickness. Models are of CS14, 15, 16, 18, 20 and 23. (B) Growth of the primary atrial septum (purple) and the secondary atrial foramen. The secondary atrial foramen only shows a trend towards narrowing (Straight line fit *P*=0.183). In grey, all myocardium belonging to the left atrium; in purple, Primary_atrial_septum_myocardium. Models are of CS14, 15, 16, 18, 20 and 23. (C) Widening of the atrioventricular canal and the distance between the interventricular septal wall and the right wall of the atrioventricular canal. In grey, AV_canal_myocardium, in orange Interventricular_septum_myocardium. Models are of CS10, 12, 14, 16, 18 and 20. (D) Closure of the interventricular foramen. In purple, Primary_atrial_septum_myocardium; in orange, the Interventricular_septum_myocardium; in grey, all other myocardium. The angle of cross-section was determined by the interventricular septum. Slices represent 10% of the total model thickness. Models are of CS 14, 15, 16, 18, 20 and 23. X-axes of the graphs are in average gestational age (days). L, left; R, right; Dm, dorsal; V, ventral. Foramina are indicated with a red arrow. *N*=1 for each time point.

The 3D reconstructions also allow for morphometric investigations (Fig. 6). Around CS16, in the sixth week of development, the primary foramen of the atrial septum closes by approximation of the mesenchymal cap mesenchyme at the leading edge of the primary atrial septum to the atrioventricular cushions (Fig. 6A). The primary foramen has never been larger than 0.1 mm^2^ (Fig. 6A). Simultaneous with the closure of the primary foramen of the atrial septum, the secondary foramen of the atrial septum opens (Fig. 6B). The volume of primary atrial septum myocardium stabilises over time, while the area of the secondary atrial foramen shows only a trend towards narrowing from 0.15 mm^2^ to 0.04 mm^2^ towards the end of the embryonic development (quadratic curve over straight line fit *P*=0.183). It is unlikely that the secondary atrial foramen narrows in development since it has to grow to approximately 45 mm^2^ perinatally (Kiserud and Rasmussen, 2001).

The opening of the atrioventricular canal in the transverse plane changes from a circular lumen to a more oval lumen with its long-axis from right to left. The canal widens linearly. Additionally, the canal shifts to the right relative to the crest of the interventricular septum (Fig. 6C). This shift of approximately 0.5 mm causes the atrioventricular canal to keep overriding the right ventricle.

The interventricular foramen is tiny from its first appearance, less than 0.1 mm^2^ (Fig. 6D). It is closed by mesenchymal tissue towards the end of the embryonic period, which, from then on constitutes the still very small membranous septum. The atrioventricular cushions play a key role in the division of the interventricular foramen in the right ventricular inlet and the left ventricular outlet. It is noteworthy that the cushions that will later form the membranous septum, at this stage are still thicker than the myocardial septum, leaving only small slits for the right and left atrioventricular channels.

## DISCUSSION

We have labelled 58 structures in ten embryonic human hearts ranging from gestational weeks 4 to 8. All structures have their own developmental stage of origin and growth rate (Supplemental materials). The observed developmental appearance of morphologically recognisable structures in the heart corresponds well with previous accounts focusing on fewer structures (Arráez-Aybar et al., 2008; Dhanantwari et al., 2009; O'Rahilly, 1971). Some detailed differences do occur, however. We distinguished the interventricular septum at CS14, slightly earlier than some (CS16) (Dhanantwari et al., 2009), though later than others (CS12) (O'Rahilly, 1971). Additionally, we observed that in our specimens the primary atrial septum and the sinuatrial valves appeared slightly later (CS14 versus CS12 and 13) (O'Rahilly, 1971). The precise staging of the human embryos is difficult and may be imprecise and may, therefore, slightly differ between studies, which could account for the reported differences in the first appearance of structures. However, at CS14 we did see the first connection of the pulmonary vein to the left atrium, which is similar to previous reports (Blom et al., 2001). Overall, most structures visibly appear around CS12-15. This period, which corresponds to the fifth week of gestation, can be considered of particular importance for the formation of the 4-chambered heart. For example, the univentricular heart, where the right ventricle is absent or only rudimentary visible (Anderson et al., 1976; Khairy et al., 2007), as well as septal defects may find their origin in this particular period of development. Growth of the heart is rapid and exponential, because it is synchronised with the exponential growth of the whole embryo (de Bakker et al., 2016), both before, during and after the morphologically important fifth week of gestation.

The fast-growing cardiac chambers show a gradual development towards adult proportions, whereas the slow-growing components of the primary heart tube become proportionally smaller (Fig. 4) (van den Berg et al., 2009). In the adult, the left and right ventricle, and the interventricular septum encompass approximately 37±6%, 19±4% and 24±4% of total myocardial mass (Hayes and Lovell, 1966). When we look at the end of the embryonic period, the chamber proportions are 32%, 30% and 7%, indicating that the right ventricle is relatively large, which is supported by previous foetal dissections and ultrasounds (Alvarez et al., 1987; Keen, 1955; Kim et al., 1992; Rowlatt et al., 1963; St John Sutton et al., 1984), and the interventricular septum lags behind in growth as can be expected at this stage (Rolo et al., 2011, 2015).

We report in this study that the formation of the trabeculae in the left atrium starts after that of the right atrium. This is in contrast to observations in mouse, where the left atrium acquires trabeculae simultaneously with the right atrium (Savolainen et al., 2009). At a comparable developmental stage (Sylva et al., 2013), both atria in the mouse are trabeculated (embryonic day 11.5-12.5) (Hoogaars et al., 2004; Savolainen et al., 2009), whereas this is not the case in human (CS14-16) (Anderson et al., 2018; Wessels et al., 2000). This may indicate that the developmental stage of onset of trabeculation is decisive for the degree of trabeculation of the adult atrial wall. There is the notion that the left atrial wall owns its smooth appearance to the incorporation of pulmonary venous myocardium (e.g. Sadler, 2006; Schoenwolf et al., 2009). Our observations, instead, suggest an important role of the late onset of trabeculation and this is entirely compatible with the previous observation that the left atrium is smooth even if no pulmonary veins connect to it (Douglas et al., 2009).

We have shown in this study that the ventricular trabecular myocardial volume increases during the entire embryonic period, which is consistent with the only other observations on trabecular volume in human embryos (Blausen et al., 1990). In our specimens, the amount of right ventricular trabeculae approached that of the left ventricle slightly later (CS23 versus CS19), possibly because the previous study included the entire ventricular septum in the measurements of trabeculae (Blausen et al., 1990). Importantly, our data together with those of Blausen et al. do not support a process of myocardial compaction, that is a decrease in trabecular myocardium by its addition to the compact wall, to occur in the embryo, as has been suggested previously (Almeida and Pinto, 2013; Sedmera et al., 2000). Although compaction appears to be well documented in chicken (Rychterová, 1971; Sedmera et al., 2000) and other animals (Hanemaaijer et al., 2019), we know of no measurements that support compaction in human. Therefore, if left ventricular non-compaction cardiomyopathy occurs, where compaction is assumed to fail (Finsterer et al., 2017; Towbin and Jefferies, 2017), it does not originate in the embryo.

We found the interventricular foramen closing between CS20 and CS23, similar to previous reports (Arráez-Aybar et al., 2008; Goor et al., 1970). The largest size of the interventricular foramen in the embryo was found to be 0.07 mm^2^, which corresponds with a foramen diameter of 0.3 mm, reported previously (Goor et al., 1970). Membranous septal defects in hearts of newborns can have diameters of 3 to 25 mm (Sharif et al., 1989), equalling 7 to 490 mm^2^. Such orders of magnitude differences in size of the foramen between the embryo and the newborn indicate that these defects should not be considered the persistence of an embryonic defect only. Similarly, the secondary atrial foramen or foramen ovale did not exceed the 0.1 mm^2^ at the end of embryonic development. It is known from ultrasounds that it increases in foetal development till an average of 0.45 cm^2^ (Kiserud and Rasmussen, 2001). This, too, indicates that the foramen itself has to expand tremendously to keep up with the physiological demands of the right-left shunt.

The rightward expansion of the atrioventricular canal was previously inferred from tracing the remodelling of the G1N2 expression domain (Wessels et al., 1992). This expansion is approximately 0.5 mm in the last week of embryogenesis, which is substantial in proportion to the heart. If the right atrioventricular junction is underdeveloped, such as in tricuspid atresia, the normal growth of the atrioventricular junction is likely perturbed (Kim et al., 2001). It is not clear to what extent the right atrioventricular junctional growth is perturbed relative to that of the left atrioventricular junction, but this could be assessed in foetuses by measurements such as we report here.

### Technical limitations

For each Carnegie stage investigated, we have included one specimen. The specimens were collected, sectioned and stained previously. Several stained sections had deformed slightly during staining procedures. Therefore, perfect alignment of these sections was not possible, which caused the fully assembled models to have a slightly striped appearance (Figshare Supplement). Also, the state of contraction of the hearts was unknown and the hearts of CS14, 15, and 16 had (partially) collapsed atria leading to noise in the luminal values (Fig. 3 and Fig. S3) and possibly in the atrial morphometric measurements (Fig. 6B). Therefore, we have only focussed on myocardial and cardiac mesenchymal volumes. Towards the in- and out-flow of the heart the extent of included sections differed. This is why the vasculature could not be labelled to the same extent for all specimens.

Given that only one specimen per stage was included, biological variation remains difficult to assess. Because most of the heart grows exponentially, as is also the case for other organs and the embryo as a whole (de Bakker et al., 2016), the increase in size with each subsequent stage we analysed is likely much larger than the biological variation in size per stage. Because we measured volumes on a range of developmental stages, each stage before and after a particular stage serves as its control. For smaller structures that undergo some level of temporary regression, such as the primary atrial septum, which develops secondary perforations, addition of more samples could substantially improve the assessment of growth. In particular, there may be substantial variation in the development of the secondary perforations as suggested by the heterogeneous appearance of primary septal defects (Patten, 1938). Because we plotted our findings on an average gestational age scale rather than a CS scale, there is some space for variation in age as well. The heart of CS15, which is slightly larger than the heart of CS16, illustrates this problem.

The embryos we used will have shrunken due to the fixation process. Previous reports have measured the shrinkage associated with 10% formalin fixation to range between 10 and 26% (Mandarim-de-Lacerda, 1991; Patten et al., 1929) and we, therefore, consider it likely that our measurements are slight underestimations. We do not know of studies that show that shrinkage is greater in some embryonic stages than in others, therefore, we expect that direction of growth and the relative growth will be correct even if the absolute values are underestimated.

## CONCLUSION

This study provides a quantitative description of the growth of the different cardiac structures recognisable in the embryonic heart. We observed exponential growth of the heart and measured the development of the septal foramina. By comparing our data with pathological reports and ultrasound investigations, we show that our quantitative description of heart development can serve as a supportive document to several fields of research and can give new insights in complex processes.

## MATERIALS AND METHODS

### Embryos

This study makes use of a human embryonic section series that was published previously (Sizarov et al., 2011a,b, 2012) so no informed consent was obtained for this investigation. The embryos were leftover material from induced abortions on social indication performed at the Gynaecology Department of the Tartu University Hospital, Estonia. They had been collected with permission of the Medical Ethics committees of the University of Tartu, Estonia, and of the University of Amsterdam, the Netherlands. The investigation conformed to the principles outlined in the Declaration of Helsinki. The embryos were fixed in 4% paraformaldehyde after collection.

After exclusion of outward abnormalities, embryos had been staged on the basis of outward appearances before they were dehydrated, immersed in butanol, and embedded in paraffin (Sizarov et al., 2011a). We included embryos of Carnegie Stage (CS) 10, 11, 12, 13, 14, 15, 16, 18, 20 and 23. Since CS does not correspond linearly to time, average ages were calculated (Sylva et al., 2013). This study did not use animal models or tissues.

### Immunofluorescent staining

The embryos had previously been sectioned at 7 (CS10, 11, 12, 13, 14, 15, 16, 18, 23) or 10 µm (CS20) thickness. Thereafter, sections that contained heart had been immunofluorescently stained with Troponin I [1 : 250; MAB1691, Chemicon or 1:250; Hytest, 4T21/2 (only on CS11, 12 and 13)] or, on alternating sections, Troponin I, SERCA2a (1:250; ab2817, Abcam) (Sizarov et al., 2010) and MF20 (produced in house after Bader et al., 1982).

### Cardiac reconstruction

With a fluorescence microscope Leica DM6000, driven by ImagePro Plus 6.2 software (Media Cybernetics), each stained section had been photographed (Sizarov et al., 2010) and was imported in Amira 6.5.0 (Konrad-Zuse-Zentrum Berlin; FEI SAS, Thermo Fisher Scientific) (Fig. 1A). The x and y values of the voxel size were set to correspond with the actual tissue size on a section. The z value corresponded to section thickness. If a section was severely damaged, it was substituted with a copy of an adjacent section.

The AlignSlices module of Amira software allows for automatic alignment of the sections without deformation correction, while permitting manual adjustments of transformation and rotation. This was done for all hearts. Next, all recognisable cardiac structures were manually segmented on each individual section (Fig. 1B). The description of all label definitions can be found in the Supplemental materials. Hereafter, with the Material Statistics tool, volumes of each labelled structure were exported. The absolute volumes and the volume growth curves of all cardiac structures can be found in Table S1 and Figs S1–S3. Three-dimensional reconstructions were made after resampling the label files. The models for CS10, 11 and 12 were resampled by averaging two voxels in x and y, CS13, 14, 15, 16, 18 and 20 were resampled by averaging four voxels in x and y and CS23 was resampled by averaging six voxels in x and y. Using the Generate Surface tool, the labels were transformed into a 3D model. With Surface View the labels of interest can be selected to be displayed (Fig. 1C). The Surface Cross Section option allows for cross-sections of the displayed labels in any plane (Fig. 1D).

For morphometric investigations, area measurements were obtained using the Polygon Selection tool in ImageJ (1.52a, Wayne Rasband, National Institutes of Health, USA). Each area was measured on the basis of a polygon with at least eight points.

In the Figshare supplement, interactive 3D pdfs of the hearts are included. The 3D models were created using the Generate Surface tool in Amira with the Smoothing option set to None. Hereafter, the Surface (.surf) file was simplified in the Simplification editor to 1,000,000 faces with the options Preserve slice structure, Fast and Create level-of-detail switched on. Hereafter, the Amira surface views were imported in Fiji (ImageJ 1.53c) in order to generate Universal 3D (.u3d) files that could be imported as 3D Object in Adobe Acrobat Pro DC (version 2020.013.20064) after clustering of labels for the navigation panel in Deep Exploration (version 6.5 CSE, Corel DESIGNER Technical Suite X5). A custom-made user interface based on javascripts is embedded in the files. The 3D pdfs can be viewed in recent versions of Adobe Reader on MS Windows or MacOS systems with javascript and playing of 3D content enabled. With the button rows on the left side of the pdf, individual or clusters of structures can be made visible (right button), transparent (middle button) or invisible (left button). By clicking and holding the left mouse button, the model will rotate when the mouse is moved. Clicking and holding the right mouse button will zoom the model when the mouse is moved. Clicking and holding both mouse buttons translates the model when the mouse is moved (Fig S4).

### Statistics

Statistical tests were performed using GraphPad Prism 8.3.0 (GraphPad Software, LLC).

## Supplementary Material

Supplementary information
