# Supplementary material for: Quantified growth of the human embryonic heart
Source: Biol Open. 2021 Feb 10;10(2):bio057059. doi: 10.1242/bio.057059 (PMC7888713; doi:10.1242/bio.057059)
Supplement: Supplementary information [file biolopen-10-057059-s1.pdf]

1. Supplemental label definitions

The following definitions are given for each of the 60 included labels:

- *IFT_myocardium*: In a linear heart tube, the myocardium at the inflow of the heart.
- *R_sinus_horn_myocardium*: Myocardialised part of the right superior caval vein entering the right atrium, part of the IFT myocardium.
- *L_sinus_horn_myocardium*: Myocardialised part of the left superior caval vein entering the right atrium, part of the IFT myocardium.
- *Atrial_myocardium*: All atrial myocardium before the formation of the primary atrial septum.
- *RA_myocardium*: Atrial myocardium to the right of the primary atrial septum minus the trabeculae, the sulcus of the secondary atrial septum, the sinuatrial valve complex, and the myocardialised part of the mesenchymal cap.
- *RA_trabecular_myocardium*: The pectinate muscles and trabeculae in the auricle of the right atrium without the septum spurium.
- *R_sinuatrial_valve*: The right sinuatrial valve leaflet as it protrudes into the atrial lumen where the systemic veins enter the right atrium.
- *L_sinuatrial_valve*: The left sinuatrial valve leaflet as it protrudes into the atrial lumen where the systemic veins enter the right atrium.
- *Septum_spurium_myocardium*: The cranial commissure of the sinuatrial valve leaflets which is more developed than surrounding trabeculae and is followed until it reaches the right atrial wall.
- *Inferior_rim_oval_fossa_myocardium*: The myocardialised mesenchymal cap and the dorsal mesenchymal protrusion, located caudal to the primary atrial septum.
- *Secondary_atrial_septum_sulcus*: Part of the right atrial myocardium that folds in on the right side of the primary atrial septum. At some stages, a protrusion similar to that of the primary atrial septum can be observed that emerges from the right atrial wall dorso-cranially.
- *Primary_atrial_septum_myocardium*: The ridge of myocardium arising in the midline from the dorso-cranial part of the common atrium as it crosses over to the atrioventricular cushions.
- *LA_myocardium*: Atrial myocardium to the left of the primary atrial septum, excluding the pulmonary vein myocardium and the trabeculae.
- *LA_trabecular_myocardium*: The trabeculae in the auricle of the left atrium.
- *Pulmonary_vein_myocardium*: Myocardium in the pulmonary venous walls, the border between it and the left atrial myocardium is drawn at the level where the left superior caval vein runs caudal to the heart.
- *AV_canal_myocardium*: Smooth atrioventricular myocardium between the atria and ventricles. In later stages it includes the myocardialised parts of the atrioventricular cushions.

Biology Open • Supplementary information

- *Myocardialised_AV_cushion*: Parts of the atrioventricular cushions that have become myocardialised.
- *Ventricular_myocardium*: The outer compact ventricular myocardium downstream of the atrioventricular canal before ventricular septation occurs. Before trabeculation, it can be separated from the atrioventricular canal myocardium and the outflow tract myocardium by its bulging appearance.
- *Ventricular_trabecular_myocardium*: The inner trabecular myocardium of the single ventricle before ventricular septation occurs.
- *LV_compact_myocardium*: The outer compact myocardium caudal to the atrioventricular canal and located to the left of the interventricular septum. Only segmented after the anterior interventricular sulcus can be recognised.
- *LV_trabecular_myocardium*: Myocardium within the left ventricular cavity.
- *RV_compact_myocardium*: The outer compact myocardium caudal to the atrioventricular canal and located to the right of the interventricular septum. Only segmented after the anterior interventricular sulcus can be recognised.
- *RV_trabecular_myocardium*: Myocardium within the right ventricular cavity.
- *Interventricular_septum_myocardium*: More densely trabeculated myocardium found adjacent to the interventricular sulcus. Because it was not possible to distinguish where the interventricular septum ends and the compact ventricular myocardium begins, an artificial cut-off at a 45 degree angle is made towards the apex of the ventricle (see Figure 1B). Cranially, the interventricular septum approaches, and ultimately connects with, the atrioventricular cushions.
- *OFT_myocardium*: Permanently untrabeculated myocardium located cranial to the ventricles and ventral to the atrioventricular canal.
- *Cardiac_jelly*: Acellular mesenchyme localised between myocardium and lumen.
- *AV_cushions*: Cellularised mesenchyme located at the level of the atrioventricular canal. By indentation it can be segmented separately from the other cushions.
- *OFT_cushions*: Cellularised mesenchyme in the outflow tract of the heart. A sharp border between it and the mesenchyme of the aorta and pulmonary artery is drawn where the semilunal valve primordia are located and the myocardium connects with the arterial wall.
- *DMP_cushion*: Cellularised mesenchyme protruding into the atria from the mesenchyme located dorsal to the heart towards the atrioventricular canal cushions.
- *Mesenchymal_cap*: Cellularised mesenchyme at the leading edge of the primary atrial septum. In later stages it connects with the atrioventricular cushions, it can still be segmented separately since the lining of the cushions show an indentation.
- *Epicardium*: Mesenchyme located on the outside of the heart. It has been reported that the epicardium covers the entire heart around CS15 (Risebro et al., 2015). On the basis of the

myocardial stained sections this one cell-layer could not be robustly differentiated from the myocardium. This is the reason that the *Epicardium* label only includes the thicker mesenchyme in the atrioventricular and interventricular groove. Similarly, we chose not to label the endocardium.

- *Veins*: Non-myocardial wall of the veins connecting to the heart before the caval veins are formed.
- *R_systemic_vein_non-myocardium*: Non-myocardial wall of the right superior caval vein.
- *L_systemic_vein_non-myocardium*: Non-myocardial wall of the left superior caval vein.
- *Inf_systemic_vein_non-myocardium*: Non-myocardial wall of the inferior caval vein.
- *Pulmonary_vein_non-myocardium*: Non-myocardial wall of the pulmonary veins.
- *Truncus arteriosus*: Non-myocardial wall, up to the pericardial reflection, of the artery connecting to the heart before outflow tract septation occurs.
- *Pulmonary_artery*: Non-myocardial wall of the pulmonary artery.
- *Aorta*: Non-myocardial wall of the aorta.
- *IFT_lumen*: Lumen surrounded by inflow tract myocardium and bordered by the sinuatrial valve leaflets.
- *R_sinus_horn_lumen*: Inflow tract lumen surrounded by the myocardial portion of the right superior caval vein.
- *L_sinus_horn_lumen*: Inflow tract lumen surrounded by the myocardial portion of the left superior caval vein.
- *IVC_lumen*: Lumen surrounded by the non-myocardialised inferior caval vein.
- *Coronary_sinus_lumen*: Inflow tract lumen bordered by right atrial lumen as surrounded by atrial myocardium and the lumen of the left sinus horn up until the point where the left superior caval veins shows a narrowing of the lumen.
- *Pulmonary_vein_lumen*: Lumen surrounded by pulmonary vein myocardium that continues into the lung mesenchyme located dorsally to the heart.
- *Atrial_lumen*: Lumen surrounded by atrial myocardium before atrial septation.
- *RA_lumen*: Lumen surrounded by right atrial myocardium. Bordered by inflow tract lumen, left atrial lumen through the primary and secondary atrial foramina and the atrioventricular canal lumen.
- *LA_lumen*: Lumen surrounded by left atrial myocardium. Bordered by pulmonary vein lumen, right atrial lumen and atrioventricular canal lumen.
- *AV_canal_lumen*: Lumen in between the atrial and ventricular lumens.
- *Ventricular_lumen*: Lumen surrounded by ventricular myocardium before ventricular septation occurs.
- *RV_lumen*: Lumen surrounded by right ventricular myocardium. Bordered by atrioventricular lumen, left ventricular lumen through the interventricular foramen, and outflow tract lumen.

- *LV_lumen*: Lumen surrounded by left ventricular myocardium. Bordered by atrioventricular lumen, right ventricular lumen, and outflow tract lumen.
- *OFT_lumen*: Lumen surrounded by outflow tract myocardium.
- *Pulmonary_artery_lumen*: Lumen surrounded by non-myocardial tissue of the pulmonary artery or by outflow tract myocardium if the outflow tract cavity had split by approximation or fusion of the outflow tract cushions.
- *Aorta_lumen*: Lumen surrounded by non-myocardial tissue of the aorta or by outflow tract myocardium if the outflow tract cavity had split by approximation or fusion of the outflow tract cushions.
- *Coronary_vasculature_lumen*: Vascular lumen found in the epicardial mesenchyme covering the heart and in parts of the outer ventricular compact walls. In more advanced stages it connects to the aortic lumen and the coronary sinus lumen.
- *Venous_lumen*: Lumen of the systemic veins not otherwise annotated.
- *Arterial_lumen*: Lumen of the systemic arteries not otherwise annotated.

Biology Open • Supplementary information

2. Supplemental figures

Figure S1: Growth curves of all myocardial labels.

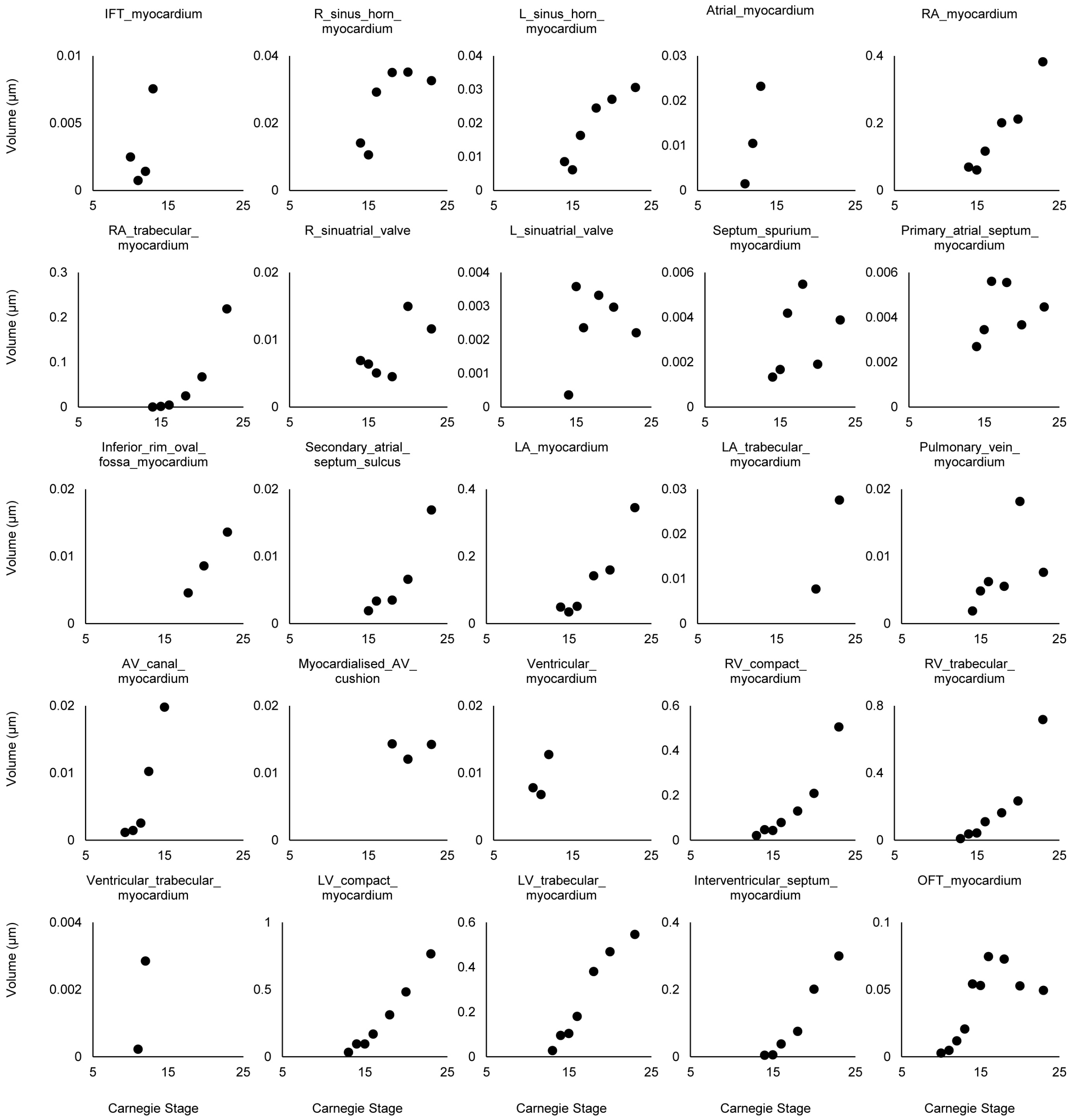

Biology Open • Supplementary information

Figure S2: Growth curves of all mesenchyme labels.

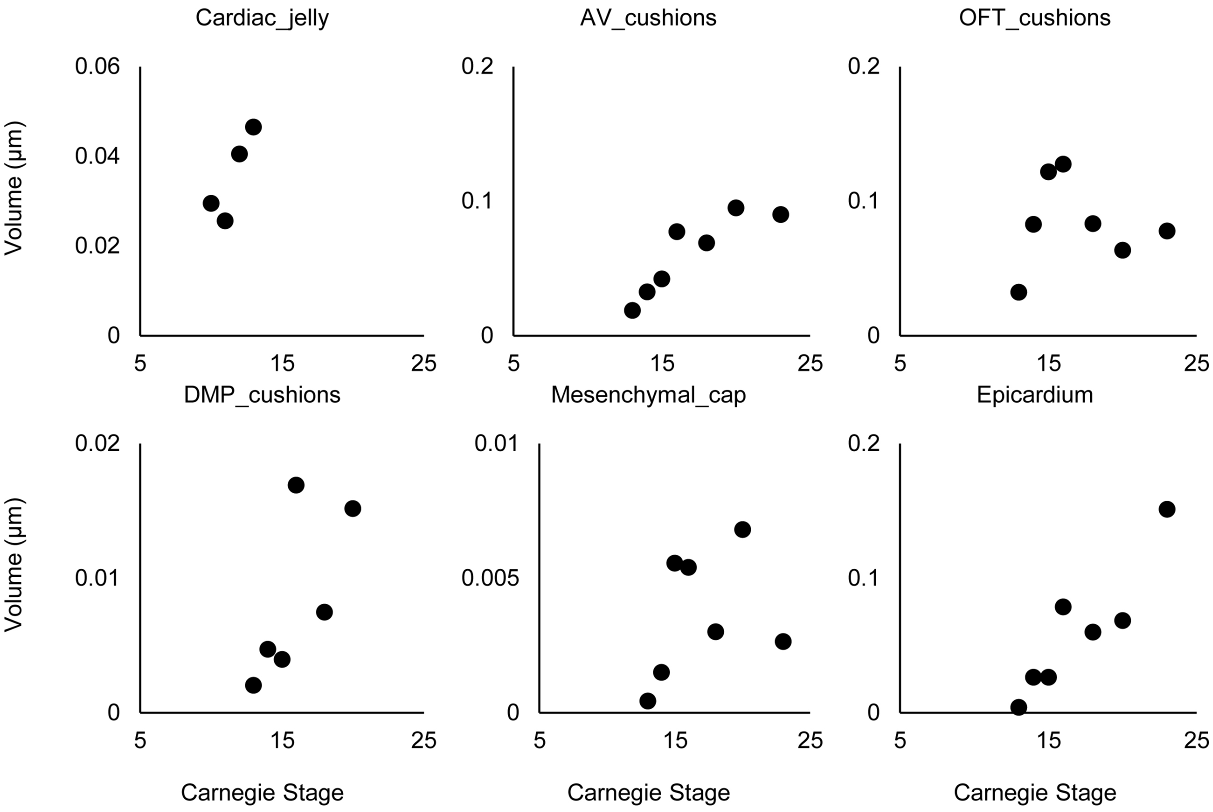

Biology Open • Supplementary information

Figure S3: Growth curves of the cardiac luminal labels.

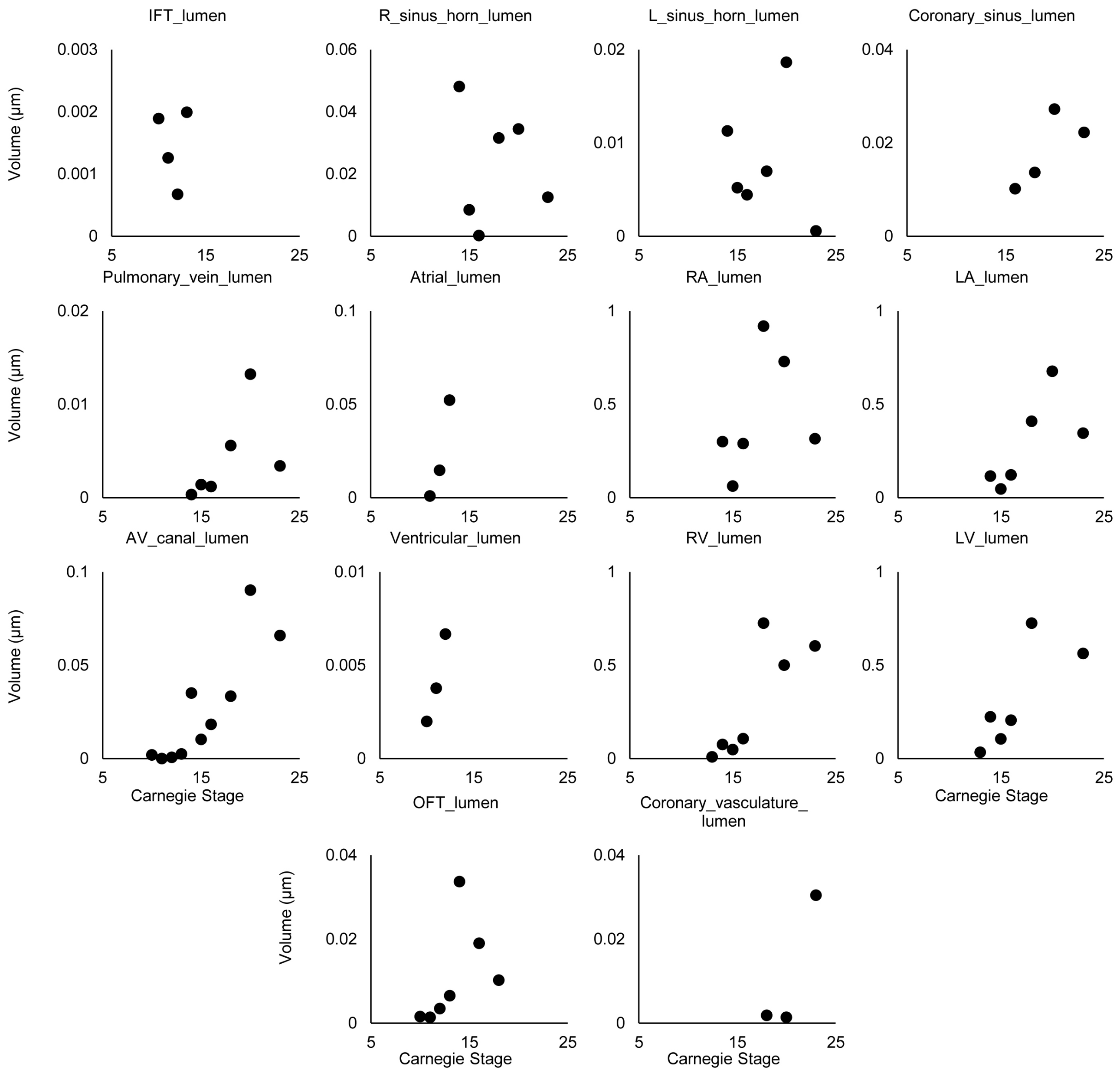

Biology Open • Supplementary information

Figure S4: Overview of a 3D pdf when opened in Adobe Acrobat Reader

Clicking on the model (1) activates it. When the left mouse button is clicked and held the model will turn when the mouse is moved. Clicking on one of the pre-set views (2) opens this view. Here, the 'Specimen' view is used. The button panel (3) allows for a pre-set selection of labels to be made visible (left button), made transparent (middle button) or invisible (right button). Clicking on a structure in the model (4) allows for an individual label to be made transparent or invisible (5). All labels can be viewed in the Model Tree option of Acrobat (6). Here all labels can be switched on or off individually.

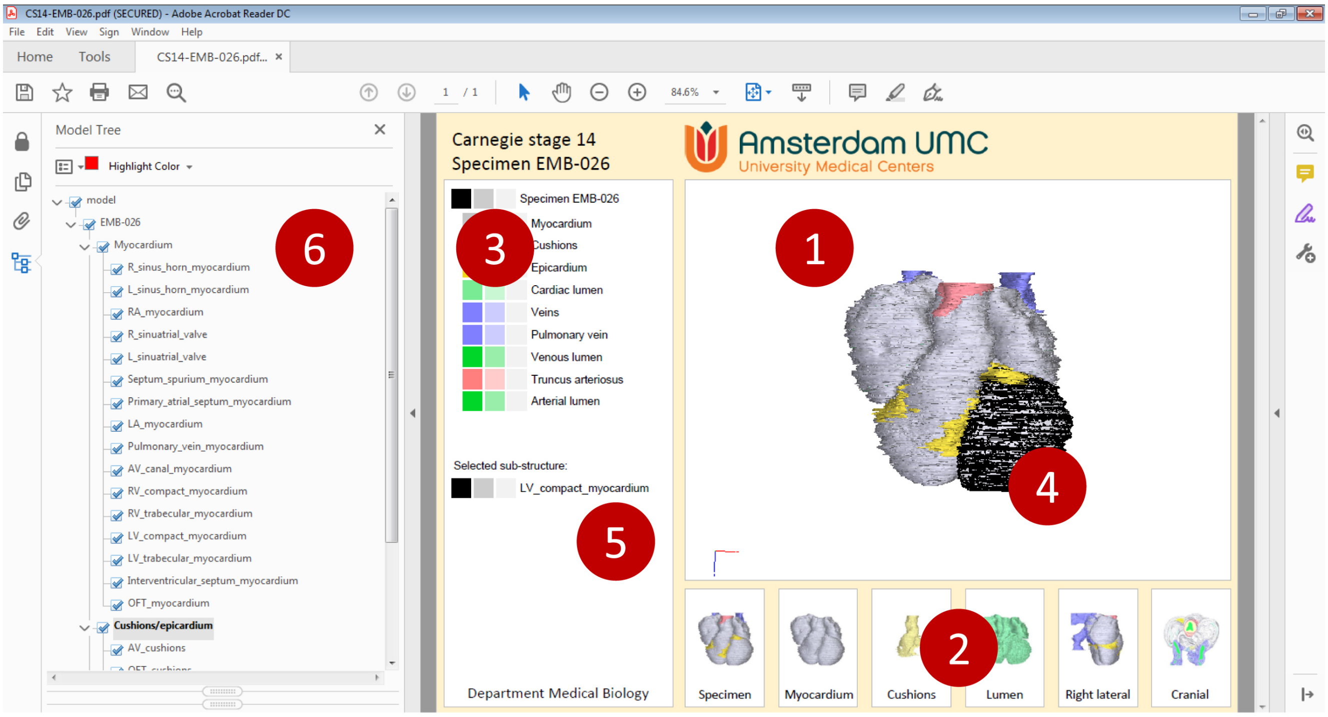

Biology Open • Supplementary information

Biology Open (2021): doi:10.1242/bio.057059 Supplementary information

4. Supplemental table

Table S1: Absolute volumes of all annotated structures.

	Absolute volumes in μm^3				
Embryo name	EMB-013	EMB-071	EMB-054	EMB-028	EMB-026
Carnegie Stage	10	11	12	13	14
Average age (days)	22.5	25	28	30	33
IFT_myocardium	2497201	739903	1398677	7556089	
R_sinus_horn_myocardium					14100356
L_sinus_horn_myocardium					8582809
Atrial_myocardium		1516262	10541870	23240604	
RA_myocardium					69542765
RA_trabecular_myocardium					
R_sinuatrial_valve					6897207
L_sinuatrial_valve					355349
Septum_spurium_myocardium					1331714
Primary_atrial_septum_myocardium					2695393
Inferior_rim_oval_fossa_myocardium					
Secondary_atrial_septum_sulcus					
LA_myocardium					48990781
LA_trabecular_myocardium					
Pulmonary_vein_myocardium					1865423
AV_canal_myocardium	1147746	1456946	2519883	10230860	20774545
Myocardialised_AV_cushion					
Ventricular_myocardium	7819816	6786204	12830403		
RV_compact_myocardium				21369088	46753205
RV_trabecular_myocardium				9350571	37735776
Ventricular_trabecular_myocardium		229039	2747513		
LV_compact_myocardium				33655555	97983754
LV_trabecular_myocardium				28442977	102796904
Interventricular_septum_myocardium					4759998
OFT_myocardium	2852732	4882911	11851916	20709122	54216267
IFT_lumen	1892717	1271167	673779	1995354	
R_sinus_horn_lumen					48107477
L_sinus_horn_lumen					11297381
IVC_lumen					23862091
Coronary_sinus_lumen					
Pulmonary_vein_lumen					355258
Atrial_lumen		964980	14810597	52361743	
RA_lumen					300208640
LA_lumen					116060349
AV_canal_lumen	1899076	52118	585855	2499906	35126046
Ventricular_lumen	1987245	3774989	6683083		
RV_lumen				9411349	75822974
LV_lumen				34287826	224195161
OFT_lumen	1538374	1365021	3371017	6522600	33663371
Aorta_lumen					
Pulmonary_artery_lumen					
Coronary_vasculature_lumen					
Cardiac_jelly	29532736	25508617	40362382	46518172	
AV_cushions				18932265	32599972
OFT_cushions				32135894	83749971
DMP_cushions				2035103	4714609
Mesenchymal_cap				423500	1494731
Epicardium				3823376	26244145
Veins	5101219	2441567	3182475	29497173	
R_systemic_vein_non-myocardium					22782253
L_systemic_vein_non-myocardium					27687460
Inf_systemic_vein_non-myocardium					14218531
Pulmonary_vein_non-myocardium					158085
Truncus arteriosus	919940	553958	6922516	909171	2485709
Aorta					
Pulmonary_artery					
Venous_lumen	8313145	1450450	4834238	25322489	29348130
Arterial_lumen	940676	289354	1583566	985428	1811175

Biology Open • Supplementary information

	Absolute volumes in µm^3				
Embryo name	EMB-059	EMB-078	EMB-006	EMB-005	EMB-084
Carnegie Stage	15	16	18	20	23
Average age (days)	36.5	39.5	46	52	58
IFT_myocardium					
R_sinus_horn_myocardium	10626775	29269408	35107893	35193448	32667207
L_sinus_horn_myocardium	6157467	16376238	24501661	27134988	30664154
Atrial_myocardium					
RA_myocardium	61049089	117071604	201349462	212157703	384682686
RA_trabecular_myocardium	1302125	4301346	24649883	67269684	218533721
R_sinuatrial_valve	6389070	5059623	4496434	14944948	11615932
L_sinuatrial_valve	3578241	2357845	3320338	2965480	2206801
Septum_spurium_myocardium	1666607	4182988	5477366	1907796	3883819
Primary_atrial_septum_myocardium	3447827	5606389	5552801	3664253	4458442
Inferior_rim_oval_fossa_myocardium		717911	4568947	8646076	13639716
Secondary_atrial_septum_sulcus	1910264	3349002	3484725	6576764	16886445
LA_myocardium	34826863	51615785	142053796	159652142	347427536
LA_trabecular_myocardium				7706725	27633867
Pulmonary_vein_myocardium	4847762	6244755	5541477	18163661	7624348
AV_canal_myocardium	19791969	24719200	28871517	39379171	44728444
Myocardialised_AV_cushion			14338816	12062444	14246668
Ventricular_myocardium					
RV_compact_myocardium	43626571	79352840	130113565	208709631	507901505
RV_trabecular_myocardium	43440175	109782357	163479598	233227221	717670673
Ventricular_trabecular_myocardium		169705030	312242564	483783386	766386592
LV_compact_myocardium	95272196	180973335	380855032	469267930	546527914
LV_trabecular_myocardium	103987702	38171830	75768496	201054571	299926892
Interventricular_septum_myocardium	6069429	74587560	72661130	52736876	49514108
OFT_myocardium	53116886				
IFT_lumen					
R_sinus_horn_lumen	8484999	235620	31579332	34489326	12586362
L_sinus_horn_lumen	5190600	4437421	6966071	18649254	564577
IVC_lumen	4331040	16110389	12941072	20430499	18480540
Coronary_sinus_lumen		10220799	13678712	27247188	22260978
Pulmonary_vein_lumen	1419205	1193169	5600930	13246288	3401984
Atrial_lumen					
RA_lumen	62656882	290844278	919070456	729471754	316623197
LA_lumen	46689003	123101744	409739334	677639656	348671487
AV_canal_lumen	10313618	18343940	33429322	90243216	65966572
Ventricular_lumen					
RV_lumen	48439447	107075740	726181158	500806519	603364629
LV_lumen	105553028	205971260	725392649	1071506512	562781927
OFT_lumen	4323917	19034818	10242664		
Aorta_lumen		2540222	4972334	26782601	40078035
Pulmonary_artery_lumen	2940968	853529	3362257	13466253	30655752
Coronary_vasculature_lumen			1873187	1372497	30472552
Cardiac_jelly					
AV_cushions	42247367	77529889	69139784	95163198	90145852
OFT_cushions	127109049	127533959	83266815	63536285	78036485
DMP_cushions	3946010	13468521	7458774	15159955	
Mesenchymal_cap	5552433	8832558	2995492	6798163	2643887
Epicardium	26115467	78604974	59871933	68322019	151146117
Veins					
R_systemic_vein_non-myocardium	20002201	39940260	35863068	6310614	69443717
L_systemic_vein_non-myocardium	11479485	29873710	46533685	13849299	65454142
Inf_systemic_vein_non-myocardium	4048204	10138971	14651790	13353531	7497588
Pulmonary_vein_non-myocardium	232790	615078	1781039	17696987	15041569
Truncus arteriosus	3414402				
Aorta		12072700	35685530	114990294	167704885
Pulmonary_artery		21613946	36129283	76443232	123289141
Venous_lumen	97445	84796213	203240001	30137520	240436610
Arterial_lumen		2407709			

Biology Open • Supplementary information
